# Preparation and characterization of tamoxifen loaded silica and NH2 functionalized mesoporous silica nanoparticles as delivery systems against MCF-7 breast cancer cells

**DOI:** 10.22038/IJBMS.2023.70152.15254

**Published:** 2023

**Authors:** Sepideh Taghavi, Mostafa Shahnani, Hasan Rafati

**Affiliations:** 1 Department of Pharmaceutical Engineering, Medicinal Plants and Drugs Research Institute, Shahid Beheshti University, Tehran, Iran; 2 Department of Phytochemistry, Medicinal Plants and Drugs Research Institute, Shahid Beheshti University, Tehran, Iran

**Keywords:** Amine-functionalized - mesoporous silica - nanoparticles (NH2-SBA-15), Breast cancer, Drug delivery, MCF-7 cells, Silica nanoparticles, Tamoxifen

## Abstract

**Objective(s):**

Controlled drug delivery using nanotechnology enhances drug targeting at the site of interest and prevents drug dispersal throughout the body. This study focused on loading a poorly water-soluble drug tamoxifen (TMX) into silica nanoparticles (SNPs) and amine-functionalized mesoporous silica nanoparticles (NH2-SBA-15).

**Materials and Methods:**

SNPs were prepared according to the Stöber method and functionalized with an amine group using 3-aminopropyl triethoxysilane (APTES) through a one-pot synthesis method to produce amine-functionalized mesoporous silica nanoparticles (NH2-SBA-15). Characterization of both nanoparticles was performed using FT-IR, FE-SEM, CHN analysis, porosity tests (BET), and dynamic light scattering (DLS).

**Results:**

The results showed an average particle size of 103.7 nm for SNPs and 225.9 nm for NH2-SBA-15. Based on the BET results, the pore size of NH2-SBA-15 was about 5.4 nm. In both silica nanoparticles, drug release at pH=5.7 was greater than that of pH=7.4. However, Tamoxifen-loaded NH2-SBA-15 (TMX@NH2-SBA-15) indicated the highest drug release in the acidic medium among TMX-loaded SNPs (TMX@SNPs), perhaps due to the high columbic repulsion in the functionalized NH2-SBA-15 nanoparticles. Regarding cytotoxicity results against MCF-7 breast cancer cell lines, both TMX@SNPs and TMX@NH2-SBA-15 nanoparticles exhibited greater cytotoxicity compared to the free TMX as a positive control. Although TMX@SNPs had a small size and high loading capacity, the cytotoxic effects were higher than those of TMX@NH2-SBA-15.

**Conclusion:**

Amine functionalization of SNPs can improve the potential activity of these nanoparticles for target therapy.

## Introduction

Cancer is a group of diseases that begins with abnormal proliferation of body cells, and sometimes cell divisions are uncontrollably proliferated and invade or spread to adjacent tissues. Despite more than ten million new cases each year, the World Health Organization estimates that the number of cancer deaths will reach 13.1 million by the year 2030 (1). One of the most common cancers among women worldwide is breast cancer (2). Conventional approaches to treat cancer include surgery, radiotherapy, and chemotherapy, where chemotherapy is often used to treat cancer. However, chemotherapy often affects healthy tissues like bone marrow and liver, which can be fatal for some cancer patients (3, 4).

Nanomedicine is the usage of nanotechnology for medical applications which has been widely developed in the last decades (5, 6). The goal of nanomedicine is to design and synthesize a variety of nano-carriers that can carry sufficient drug loads to eventually cross physiological barriers and deliver the drug to the target sites and finally, cure diseases (7). Therefore, it is desirable to develop chemotherapeutics to target cancer cells passively or actively for both purposes of reducing the adverse effects and improving the therapeutic effect. To this end, several nano-delivery systems have been developed in the past few years that target tumors and increase therapeutic efficacy, including polymeric nanocarriers (8, 9), liposomes (10), nano-size lipids (11, 12), and inorganic carriers (13).

 Nanoparticles have unique biological properties due to their small size and large surface area, which allows them to load various therapeutic agents such as drugs, and carry them to the target tissues (1). In drug delivery, drugs are either dissolved, entrapped, encapsulated, or attached to a nanoparticle matrix (14). Drug-loaded nanoparticles may release the drug in response to various stimuli, including pH, which is among the most important internal stimuli, since some diseases such as cancer, exhibit pH changes in the evolution. The internal environment inside the tumor cells is more acidic than the surrounding environment (15, 16). Thus, when drug-loaded nanoparticles internalize the tumor cells, the drug will be released by the pH changes (17).

Nanocarriers for drug delivery can be classified into two major groups, organic and inorganic. In recent years, inorganic nanoparticles have been used for therapy and imaging due to their interesting advantages, such as large surface area, high drug loading capacity, improved drug bioavailability, controlled drug release, and low toxic side effects (1). Mesoporous silica nanoparticles are a subgroup among the inorganic nanoparticles that are gaining increasing popularity in drug delivery (18, 19). It demonstrates particular characteristics such as significantly lower cytotoxicity at high concentrations when compared to other inorganic nanomaterials. In addition, it can be considered a reservoir that can load a large quantity of drug molecules in the pores (19). Besides, mesoporous silica nanoparticle has been the focus of many applications, including catalysis, drug delivery, chromatography due to its large surface area, and inert chemical reactivity, biocompatibility, and also low prices (20-26). Mesoporous silica particles are made by the condensation of sodium silicate or silicon alkoxides around a surfactant as a template. The synthesis of these particles depends on a variety of conditions, including pH conditions, ionic strength, temperature, and surfactant morphology, which directly affect the pore size, pore volume, specific surface area, and wall thickness (27). The Mobil Crystalline Material (MCM) was designed for the first time in 1992 by the Mobil Company (27). The MCM materials contain various types of particles called MCM-41, MCM-48, and MCM-50, which exhibited hexagonal, cubic, and lamellar mesostructures, respectively (28). The nanoparticles made of mesoporous silica compared to liposomes and dendrimers are more resistant to external responses like degradation and mechanical stress because of the presence of strong Si-O bond, which is the reason for no need for external stabilizers in the synthesis of this type of nanoparticle (29, 30). Due to the high bonds of Si-OH on the surface of the mesoporous silica particles, these are easily functionalized and modified (31). For instance, when the surface of the nanoparticle is functionalized with amine groups and the drug also has an amine group in its structure, despite the acidic environment inside the tumor, it can lead to columbic repulsion between the positive charges, which triggers drug release. Synthesis of silica nanoparticles was first reported by Stöber in 1968 (32). According to the Stöber method, monodisperse silica particles have a spherical shape with diameters ranging from 50 nm to 2 µm and were successfully made in a mixture of water, alcoholic solvent, ammonia, and tetra alkoxysilane. In 1998, a mesoporous substance called Santa Barbara (SBA), was first reported by Zhao *et al*. (33). SBA-15 has well-ordered hexagonal mesostructures, and its pore size can be adjusted from 2 to 50 nm, which is a key advantage of this porous silica compared to MCM materials and expands the number of applications. SBA is synthesized using non-ionic surfactants such as polypropylene oxide and polyethylene oxide, which are regarded as pore-directing agents in an acidic medium (27).

Mesoporous silica nanoparticles are widely used for drug delivery and their loading procedure will be based on physical or chemical adsorption. In contrast, the loading procedure in silica nanoparticles will be based on encapsulation or conjugation (7, 34, 35). The cargo release profile from mesoporous silica nanoparticles can be controlled either by using the “gatekeeper” strategy or by modifying the inner surface of the pores to control the binding affinity with drugs, whereas the release profile of the cargo from silica nanoparticles can be controlled either by chemical bonds or the degradation of silica matrix (7, 35). 

In the present work, we will focus on inorganic nanocarriers and more specifically mesoporous and nonporous (solid) silica nanoparticles. These two kinds of silica nanoparticles were synthesized. Tamoxifen Citrate (TMX) as a highly hydrophobic anticancer drug has been used for years as the antiestrogen treatment of advanced or metastatic breast cancer (36-38). TMX loading procedure on both silica nanoparticles was performed and consequently, the drug release profile from both kinds of silica nanoparticles was studied. Finally, the cytotoxicity of TMX@SNPs and TMX@NH_2_-SBA-15 was investigated on the MCF7 cancer cells.

## Materials and Methods

***Materials and Instruments***

Ethanol (Kimia Alcohol Zanjan Company, Iran, 96%), Tetraethyl orthosilicate (TEOS) (Merck, Germany), Pluronic P123 (Sigma Aldrich, Germany), Ammonium acetate, Acetic acid, and acetonitrile HPLC grade from (Biochem chemopharma, France), Tamoxifen citrate (Iran Hormone Company, Iran), methanol HPLC grade (CARLO ERBA, France), Ammonia solution (Chem-lan NV, Belgium, 25%), 3-Aminopropyl triethoxysilane (APTES) (Exir Wien, Austria) HCl (ERBA pharma,37%), NaCl (Biochem), KCl, Na_2_HPO_4_ and KH_2_PO_4 _from (Merck), were obtained as purchased. The deionized and purified water was used throughout all experiments by using a Millipore Milli-Q system (Millipore Direct-Q UV, Molsheim, France). The nanoparticles were freeze-dried using a freeze dryer Alpha 1-2 LD plus (Germany) and evaluated in terms of shape using a Hitachi FE-SEM (s4160, Japan). To confirm the porous structure of NH_2_-SBA-15, nitrogen adsorption-desorption isotherm was measured on a Belsorp mini II (Bel, Japan). HPLC analysis of the samples was performed using Knauer (Berlin, Germany).

***Synthesis of silica nanoparticles***

Silica nanoparticles were prepared using the Stöber synthesis (32). In a typical procedure, 25 ml water was stirred into 23.3 ml ethanol for 10 min. Then 1 ml of TEOS was added into the mixture under continuous stirring for 20 min. Finally, 25 ml NH_4_OH (28%) as a catalyst, was added into the same solution and stirred for 2 hr. As a result, a white turbid suspension was obtained (39) and freeze-dried.

***Synthesis of NH***_2_ ***- SBA-15 silica nanoparticles through the one-pot method***

The preparation of amine-functionalized mesoporous silica nanoparticles (NH_2_-SBA-15) was performed using the reported protocol (40), i.e., 1 ml TEOS was introduced into a round-bottom flask containing 14.5 ml water, 800 µl (HCl 2M), and 25 mg triblock copolymer Pluronic P123 and mixed well at 1100 rpm and 40 °C for 1 hr. Then, under nitrogen atmosphere, 120 µl APTES was poured, slowly. The resultant mixture was stirred for another 20 hr under the same condition. Then the mixture was transferred to an Erlenmeyer flask for particle growth and placed in an oven for 24 hr at 100 °C. Finally, the resultant mixture was freeze-dried.

***Removing triblock copolymer pluronic P123 from NH***_2_ ***-SBA-15***

The freeze-dried powder was boiled in ethanol for 5 hr. Finally, it was centrifuged several times and the precipitate was placed into an oven at 40 °C for 6 hr.

***Cell culture***

Cell culture studies were performed using the human breast cancer MCF-7 cell line, which was obtained from the National Cell Bank of Iran (NCBI). Cells were grown in RPMI 1640 medium (Gibco, USA) supplemented with 10% fetal bovine serum (Gibco) containing penicillin (100 units/ ml) and streptomycin (100 mg/ml) at 37 °C in a humidified atmosphere of 5% CO_2_. The medium was changed twice a week and trypsin-EDTA (Sigma) 25% w/v was utilized for cell´s detachment. Cell number and viability were ascertained using trypan blue (Sigma) dye and a hemocytometer (41).

***In-vitro cellular cytotoxicity***

*In vitro* cytotoxicity of blank SNPs, TMX@SNPs, and TMX@NH_2_-SBA-15 were determined by MTT assay and compared to that of the free TMX treated group in triplicates**. **TMX was dissolved in DMSO and serially diluted in a culture medium**. **The MCF-7 cells (7 ×10^3^ cells) were seeded in 96-well plates with 200 µl of growth medium per well and cultured at 37 °C in a humidified 5% CO_2_ air for 24 hr. For treatment, free TMX stock solution was prepared in the growth medium (containing 0.1% DMSO) and was further serially diluted with the growth medium. Different dilutions of samples were prepared at the same concentration of free TMX. Then, the media was replaced by fresh media containing the above-mentioned samples, and cells were incubated for 24, 48, and 72 hr. Then, 10 µl of MTT solution at a concentration of 5 mg/ml was added to each well, and cells were incubated for another 3 hr. During incubation, the MTT solution was reduced by the enzyme succinate dehydrogenase (as illustrated in Scheme1) which produced insoluble formazan purple crystals. For this purpose, 100 µl of DMSO was added to each well to dissolve the formed formazan crystals (41). The absorbance values of the samples were measured at 490 nm using a microplate reader (Biotech, ELx800, USA). Finally, the cell viability (%) was calculated as follows (42):

Cell viability (%) = $\frac{\mathrm{A490}(\mathrm{sample})}{\mathrm{A490}(\mathrm{control})}\times 100$

(1) Equation

***Preparation of Tamoxifen loaded SNPs and NH***_2_ ***-SBA-15 (TMX@SNPs and TMX@NH***_2_ ***-SBA-15)***

To this end, 30 mg of SNPs and NH_2_-SBA-15 were added to a TMX solution in methanol at a concentration of 830 ppm and stirred overnight at room temperature. The resulting suspension was centrifuged at 12000 rpm for 15 min. Finally, the supernatant was collected as unloaded drug and evaluated by HPLC. Afterward, the precipitate was washed thoroughly with methanol then as mentioned above, the same procedure was repeated to collect the supernatant (43, 44) this amount also was added to the previous value, and the total considered as unloaded drug.

The loading efficiency and loading capacity of TMX were calculated as follows:

%Loading efficiency=primary drug-unloaded drug primary drug×100

(2) Equation

$\%\mathrm{Loading capacity}=\frac{\mathrm{Wloaded drug}}{\mathrm{W loaded drug}+\mathrm{W nanoparticles}}\times 100$

(3) Equation

**5.7**) containing Tween 80 (0.5% w/v) and PBS (pH 7.4) containing Tween 80 (0.5% w/v). Particularly, pH values of tumor environments. Additionally, due to the poor aqueous solubility of TMX, Tween 80 (0.5% w/v) was added to the release medium (43).

8.5 mg of TMX@SNPs and TMX@NH_2_-SBA-15 were dispersed in both media, separately. Then, they were placed in a shaker-incubator at 37 °C and 150 rpm. At predetermined time intervals up to 3 days, 1 ml of the suspension was withdrawn and replaced with an equal volume of each fresh medium, and finally, it was centrifuged at 12000 rpm for 15 min. The supernatant was collected for drug measurement using HPLC to evaluate the amount of released Tamoxifen (44).

**Figure 1:**
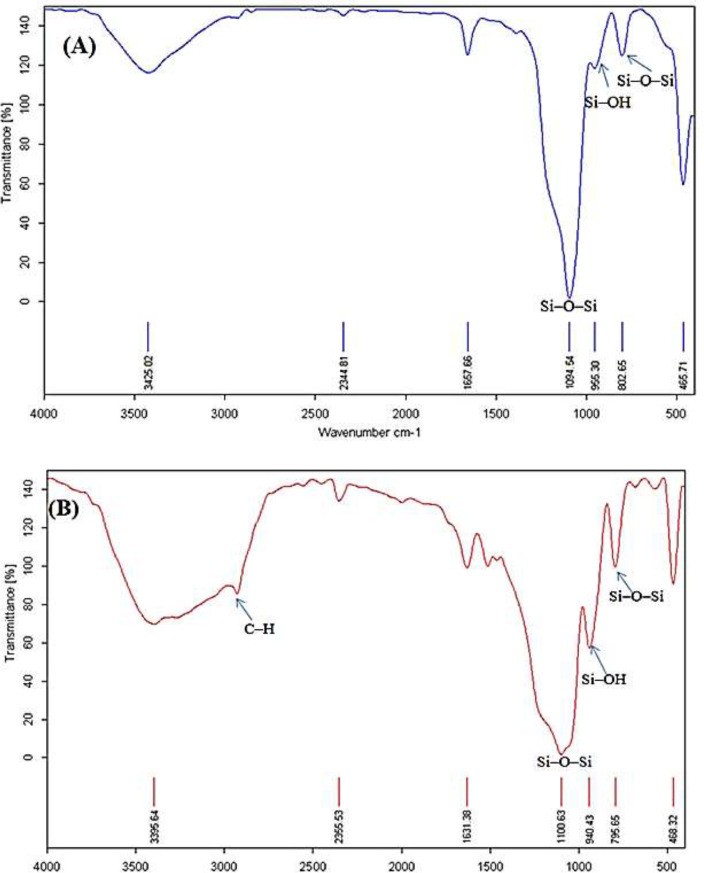
FT-IR spectra of SNPs and NH2-SBA-15 after extraction with ethanol

SNPs: Silica nanoparticles; NH2-SBA: Amine-functionalized mesoporous silica nanoparticles

**Table 1:** CHN Elemental analysis of NH2 functionalized SBA-15 nanoparticles

Sample	Carbon (wt%)	Hydrogen (wt%)	Nitrogen (wt%)
NH_2_-SBA-15	2.51	11.50	1.27

**Figure 2:**
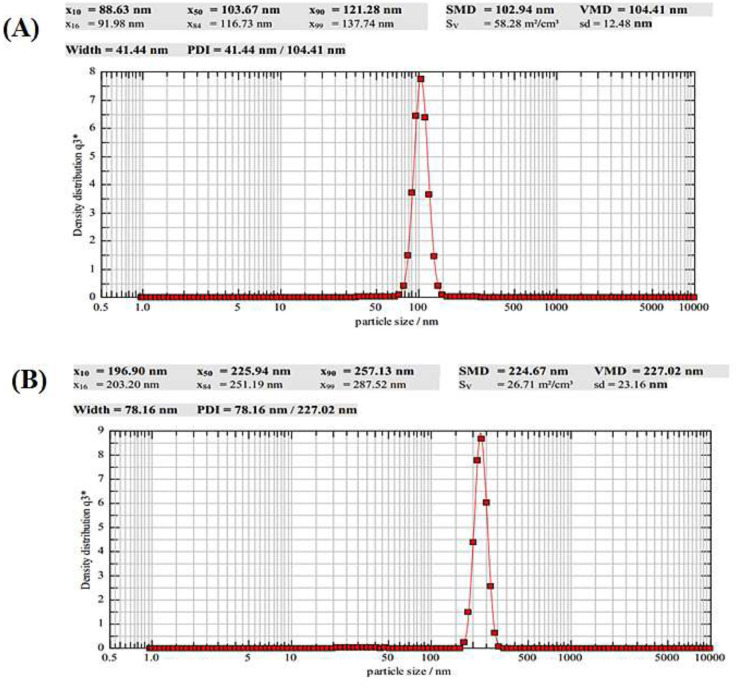
The average particle size of SNPs (A) and NH2-SBA-15 (B)

SNPs: Silica nanoparticles; NH2-SBA-15: Amine-functionalized mesoporous silica nanoparticles

**Figure 3:**
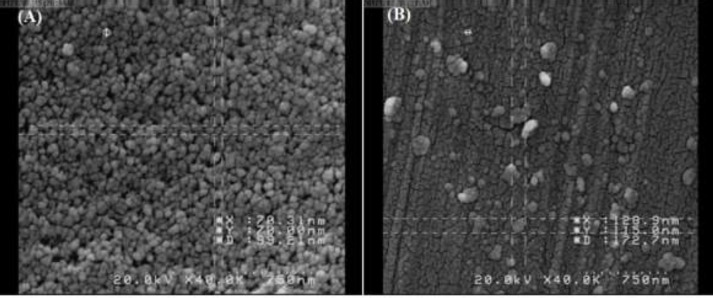
FE-SEM image of SNPs (A) and NH2-SBA-15 (B) for visual control of nanoparticles

SNPs: Silica nanoparticles; NH2-SBA-15: Amine-functionalized mesoporous silica nanoparticles

**Figure 4:**
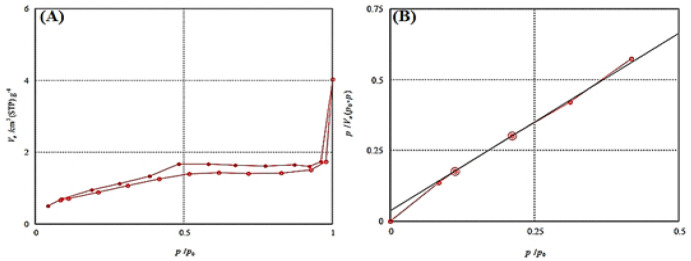
Nitrogen adsorption/ desorption isotherm (A), and BET plot (B) to confirm mesoporous structure of NH2-SBA-15

BET: Porosity tests; NH2-SBA-15: Amine-functionalized silica nanoparticles

**Table 2:** Loading amount of formulated nanoparticles

Nanoparticles	% Loading capacity (DLC %)	% Loading efficiency (LE %)
SNPs	0.73	1.7
NH_2_-SBA-15	0.26	0.55

SNPs: Silica nanoparticles; NH2-SBA-15: Amine-functionalized mesoporous silica nanoparticles

**Figure 5:**
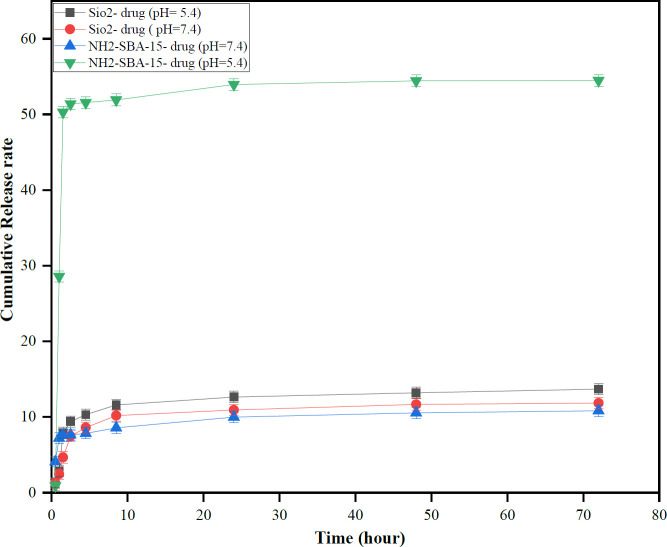
Drug release profile from SNPs and NH2-SBA-15 SNPs for pH-dependent release of TMX from nanoparticles

SNPs: Silica nanoparticles; TMX: Tamoxifen; NH2-SBA-15: Amine-functionalized mesoporous silica nanoparticles

**Figure 6:**
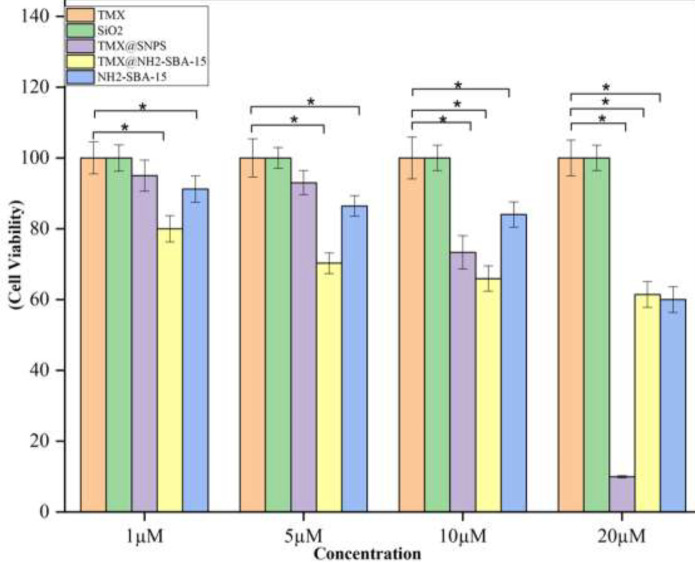
Cytotoxicity of TMX@SNPs, TMX@NH2-SBA-15 compared to the free TMX, Free SNPs, and free NH2-SBA- 15 nanoparticles against MCF-7 cells

Data represent mean±SD (n=3). Data were analyzed using one-way ANOVA. Significant difference was considered at *P*<0.05* compared to the control (TMX)

SNPs: Silica nanoparticles; NH2-SBA-15: Amine-functionalized mesoporous silica nanoparticles; TMX: Tamoxifen

## Results

***Characterization***

Following the synthesis of SNPs, characterization of the synthesized samples was carried out using various techniques including FT-IR spectroscopy, DLS, FE-SEM, CHN analysis, and Nitrogen Adsorption-Desorption Isotherm. The pore volume and pore size were determined using the BJH (Barrett–Joyner–Halenda) analysis, and the surface area was determined using the BET (Brunauer–Emmett–Teller) method. To quantify data from drug release, HPLC was performed using Knauer (Berlin, Germany) (Figure 1). The FT-IR spectra were used to analyze the chemical groups on the surface of the material, which here demonstrated the successful preparation of SNPs and NH_2_-SBA-15. As illustrated in Figure 1A, the signals at 955, 1,094, and 802 cm^-1^ correspond to the stretching vibration of Si–OH, and asymmetric and symmetric stretching vibrations of Si–O–Si, respectively. The broad bond observed at 3425 cm^-1^ is attributed to the O-H group. A small signal at 1657 cm^-1^ is ascribed to the bending vibration of O–H. Additionally, the signal exhibited the Si-O-Si bond-bending mode at 465 cm^-1^ (45). Accordingly, these signals are particularly representative of SNPs.

 The FT-IR spectrum after extraction with ethanol confirmed the presence of the amine group on NH_2_-SBA-15 (Figure 1B). The broad peak at 3519 cm^-1^ is related to the N–H group and the peak at 1460 cm^-1^ is described by the bending vibration of N–H. The bands observed about 2900 cm^-1^ correspond to the C–H stretching vibration due to hydrocarbon chains (triblock copolymer Pluronic P123), which are used in the synthesis of NH_2_-SBA-15. As shown in Figure 1B, there is no signal at 2900 cm^-1^ due to the removal of the surfactant in the extraction step with ethanol. In some reports, the observed distinguished absorption band in 2980 cm^-1^ is C-H stretching bands.

***Elemental analysis***

In addition to the FT-IR results spectrum that qualitatively confirmed the presence of the amine group on the surface of NH_2_-SBA-15, CHN elemental analysis was used to provide further evidence for the presence of amine groups on the surface of SBA-15, as illustrated in Table 1.

***Nanoparticles size measurement***

The mean particle size and size distribution of both nanoparticles were measured using dynamic light scattering (DLS). In this test, deionized water was employed to dilute the samples in order to achieve droplets count ranging from 200 to 2000 kCPS. As can be observed (Figure 2A and B), the SNPs and NH_2_-SBA-15 exhibited an average size of 103.7 nm and 225.9 nm with a particle size distribution (PDI) of 0.15 nm and 0.11 nm, respectively, which shows monodispersed and homogenous size distribution of the nanoparticles.

***FE-SEM images of nanoparticles***

FE-SEM images of SNPs and NH2-SBA-15 synthesized by the Stöber method were provided in (Figures 3A and B). The images show SNPs and NH2-SBA-15 have nearly spherical morphology with an average size of about 99 nm and 170 nm, respectively. These results are in line with the DLS results since DLS measures the hydrodynamic diameter of particles, which is larger than that of solid particles. 

***N***_2_ ***adsorption–desorption analysis***

As shown in Figure 4A, the vertical axis represents the amount of gas that enters the pores of NH_2_-SBA-15, and the horizontal axis represents the ratio of relative pressure in equilibrium with saturated pressure in the cavities. From the results, it can be seen that as the pressure increases, the absorption diagram moves upwards. According to IUPAC, the N_2_ adsorption–desorption isotherms of NH_2_-SBA-15 fitted a type IV isotherm (Figure 4A), and the existence of hysteresis H1 belongs to the mesoporous material where the particles have 2 to 50 nm pore size and tubular cavities (46). The specific surface area and pore size can be calculated by solving a series of equations (47). As shown in Figure 4B, the specific surface area is 337 m^2^/g and the average pore size is about 5.4 nm.

***TMX loading on nanoparticles***

A ratio of 1:3 of drug: carrier was used in the drug loading procedure to compare the loading capacity of SNPs and NH_2_-SBA-15 nanoparticles (48). As can be seen in Table 2, there is a reverse relationship between drug loading capacity (DLC) and loading efficiency (LE), and as the drug/carrier ratio increases, the DLC% increases while the LE% decreases.

As shown in Table 2, DLC% in SNPs is greater than NH_2_-SBA-15 because the size of silica nanoparticles (103.67 nm) is smaller than NH_2_-SBA-15 (225.94 nm). Therefore, the surface area of SNPs provided high drug loading capacity compared to NH_2_-SBA-15 nanoparticles, hence the DLC% was increased. There could be another explanation for the higher loading capacity because of the lower size of SNPs, which provides much more free hydroxyls group than NH_2_-SBA-15, capable of forming hydrogen bonds with TMX.

***TMX release study***

Figure 5, illustrates the percentage of TMX release profile from samples in phosphate buffer saline (PBS) at pH = 7.4 and pH =** 5.7** containing 0.5% Tween 80 within 72 hr. TMX can be adsorbed onto the surface of both types of silica nanoparticles by hydrogen bonding. In fact, the hydroxyl groups present on the surface of both silica nanoparticles should be the site of hydrogen bonding with the amine groups in TMX; Also, free amine groups in the NH_2_-SBA-15 are the reaction site of hydrogen bonding with the amine groups present in TMX.

As shown in Figure 6, the drug release profile demonstrates pH-dependent behavior and the burst effect was observed in both silica nanoparticles because the highest initial release was due to the adsorbed drug molecules on the surface of both silica nanoparticles. Furthermore, absorption of TMX in NH_2_-SBA-15 is much stronger than in silica nanoparticles because the interaction between TMX with the amine group in the NH_2_-SBA-15 is stronger than in hydrogen groups in silica nanoparticles (49). TMX release from TMX@NH_2_-SBA-15 at pH = **5.7** and 37 °C, showed the highest pH dependence behavior compared to the other samples. Therefore, in the acidic environment, a high columbic repulsion from the positive silica surfaces (protonated NH_2_-SBA-15) and the positively charged TMX was produced to promote the burst release of adsorbed TMX from NH_2_-SBA-15 (50).

***Inhibitory effect against tumor cells***

As shown in Figure 6, TMX@SNPs, TMX@NH_2_-SBA-15, and NH_2_-SBA-15 showed noticeable cytotoxicity against the MCF-7 cells compared to TMX (as a positive control) and SNPs at all concentrations. The results may be explained by the possible TMX resistance of the cells and non-toxicity of SNPs at all concentrations up to 20 µM. However, once TMX is loaded in SiO_2_ nanoparticles and presented in nanoparticulate form, increased surface loading may amplify the anticancer activity of TMX. However, once TMX is loaded onto the surface modified TMX@NH_2_-SBA-15 nanoparticles, the surface modification may hinder the interaction of TMX with the cancer cells, and the observed toxicity could be due to the inherent toxicity of surface-modified nanoparticles.

## Discussion

The purpose of this study was to demonstrate that inorganic nanocarriers such as SNPs and NH_2_-SBA-15 could be used as a drug delivery system to deliver TMX as a hydrophobic anticancer agent. Loading TMX into the SNPs and NH_2_-SBA-15 has not been reported until today. There are many silanol groups on the surface of silica materials that can be converted into other groups through chemical reactions (51). In the present work, SNPs and NH_2_-SBA-15 were synthesized and characterized using FT-IR spectroscopy, DLS, FE-SEM, CHN analysis, and Nitrogen Adsorption-Desorption Isotherm.

The FT-IR results of this study were consistent with the previous results (52). SBA-15 was further analyzed by CHN elemental analysis to demonstrate the presence of amine groups. This result was in agreement with the other reports in which the aminopropyl-functionalized SBA-15 mesoporous materials (NH_2_-SBA-15) with different lengths of channeling pores and aminopropyl loadings up to 2.6 mmol g^-1^were synthesized by one-pot co-condensation of tetraethyl orthosilicate (TEOS) and aminopropyltrimethoxysilane (APTMS) with the aid of appropriate amounts of Zr (IV) ions and NaCl (53). The mean particle size and size distribution of both nanoparticles were measured by using dynamic light scattering (DLS) and the result was depicted in Figure 2. The FE-SEM images show SNPs and NH_2_-SBA-15 have nearly spherical morphology with an average size of about 99 nm and 170 nm, respectively. These results are also in agreement with the other reports presented by Lou* et al., *showing the spherical morphology of the nanoparticles prepared by the same method (54). The N_2_ adsorption-desorption analysis was used to determine the pore size of the NH_2_-SBA-15 silica nanoparticles. Mesoporous materials have pore structures that play an important role in their physical properties. A nitrogen isotherm method can be used to determine the porous structure and textural parameters of porous materials. NH_2_-SBA-15 specimens showed a type-IV hysteresis loop with type H1 at a relative pressure of P/Po = 0.5–1, which is characteristic of mesoporous structures as shown in Figure 4a (55, 56).

Additionally, drug loading capacity is an essential property of an ideal drug delivery system. The amount of a drug that can be loaded onto a nanocarrier is known as its loading capacity (57). From Table 2, DLC% in SNPs is much higher than NH_2_-SBA-15 and there are two reasons behind this. Firstly, due to their smaller size, smaller particles have a higher loading capacity. This means they have more space to carry drugs, increasing their loading capacity (58). Secondly, the lower size of SNPs provides more free hydroxyl groups that are capable of forming hydrogen bonds with TMX.

In the next step, HPLC chromatography was used to measure drug release and the results are depicted in Figure 5. The release profiles of the targeted silica nanoparticles were investigated at a PBS buffer with two different pH values of 5.7 and 7.4 to compare with each other. Initial burst release can be attributed to TMX molecules on silica nanoparticle surfaces. The same result was observed in Sedigheh *et al. *(58). Furthermore, the drug was released faster from the NH_2_-SBA-15 nanocarrier in an acidic medium than in a neutral one. A faster release is caused by the repulsion between the positively charged TMXs and the protonated NH_2_-SBA-15 surfaces.

Finally, in order to determine the effects of these drug-nanocarriers, drug-free carriers, and TMX as a positive control on breast cancer cells, MCF7 cells were cultured and MTT assays were conducted. The results are shown in Figure 6. So, these results show that in the presence of both silica nanoparticles as nanocarriers, MCF-7 cancer cells are less likely to survive than bare TMX. A similar study also shows that in the presence of synthesized targeted MMNPs nanocarrier, MCF-7 cancer cells are less likely to survive than DOX alone (57).

## Conclusion

The results showed that for both types of silica, drug release at pH= **5.7** is greater than pH= 7.4. Drug release studies indicated that TMX@NH_2_-SBA-15 has the highest drug release in the acidic medium among the other nanoparticles, probably due to a high columbic repulsion between drug and amine groups. Overall, based on the results, both prepared nanoparticles exhibited higher toxicity than free TMX as a positive control, due to the increased solubility of TMX in the vicinity of the MCF-7 cells or inherited toxicity of surface-modified TMX@NH_2_-SBA-15.

## Authors’ Contributions

S T performed experimental research, data collection, and writing. M S performed experimental research and data analysis. H R contributed by project management, supervision, and data analysis, and edited the manuscript.

## Conflicts of Interest

None.

## References

[1] Senapati S, Mahanta AK, Kumar S, Maiti P (2018). Controlled drug delivery vehicles for cancer treatment and their performance. Signal Transduct Target Ther.

[2] Waks AG, Winer EP (2019). Breast cancer treatment: A review. JAMA.

[3] Debela DT, Muzazu SGY, Heraro KD, Ndalama MT, Mesele MW, Haile DC (2021). New approaches and procedures for cancer treatment: Current perspectives. SAGE Open Med.

[4] Li Z, Tan S, Li S, Shen Q, Wang K (2017). Cancer drug delivery in the nano era: An overview and perspectives (Review). Oncol Rep.

[5] Kim BY, Rutka JT, Chan WC (2010). Nanomedicine. N Engl J Med..

[6] Ferrari M (2005). Cancer nanotechnology: Opportunities and challenges. Nat Rev Cancer.

[7] Tang L, Cheng J (2013). Nonporous silica nanoparticles for nanomedicine application. Nano Today.

[8] Kumar S, Singh S, Senapati S, Singh AP, Ray B, Maiti P (2017). Controlled drug release through regulated biodegradation of poly(lactic acid) using inorganic salts. Int J Biol Macromol.

[9] Shim MS, Kwon YJ (2012). Stimuli-responsive polymers and nanomaterials for gene delivery and imaging applications. Adv Drug Deliv Rev.

[10] O’Brien ME, Wigler N, Inbar M, Rosso R, Grischke E, Santoro A (2004). Reduced cardiotoxicity and comparable efficacy in a phase III trial of pegylated liposomal doxorubicin HCl (CAELYX/Doxil) versus conventional doxorubicin for first-line treatment of metastatic breast cancer. Ann Oncol.

[11] Dong Y, Eltoukhy AA, Alabi CA, Khan OF, Veiseh O, Dorkin JR (2014). Lipid-like nanomaterials for simultaneous gene expression and silencing in vivo. Adv Healthc Mater..

[12] Mo R, Jiang T, Gu Z (2014). Recent progress in multidrug delivery to cancer cells by liposomes. Nanomedicine (Lond).

[13] Gu FX, Karnik R, Wang AZ, Alexis F, Levy-Nissenbaum E, Hong S (2007). Targeted nanoparticles for cancer therapy. Nano Today.

[14] Swami A, Shi J, Gadde S, Votruba AR, Kolishetti N, Farokhzad OC Nanoparticles for Targeted and Temporally Controlled Drug Delivery Multifunctional Nanoparticles for Drug Delivery Applications: Imaging, Targeting, and Delivery. Boston, MA: Springer US.

[15] Lee ES, Gao Z, Bae YH (2008). Recent progress in tumor pH targeting nanotechnology. J Control Release.

[16] Gethin GT, Cowman S, Conroy RM (2008). The impact of Manuka honey dressings on the surface pH of chronic wounds. Int Wound J.

[17] Casey JR, Grinstein S, Orlowski J (2010). Sensors and regulators of intracellular pH. Nat Rev Mol Cell Biol.

[18] Baek S, Singh RK, Khanal D, Patel KD, Lee EJ, Leong K (2015). Smart multifunctional drug delivery towards anticancer therapy harmonized in mesoporous nanoparticles. Nanoscale.

[19] Mai WX, Meng H (2013). Mesoporous silica nanoparticles: A multifunctional nano therapeutic system. Integr Biol (Camb).

[20] Slowing II, Trewyn BG, Lin VS (2007). Mesoporous silica nanoparticles for intracellular delivery of membrane-impermeable proteins. J Am Chem Soc.

[21] Sayari A (1996). Catalysis by crystalline mesoporous molecular sieves. Chem Mater.

[22] Hartmann M (2005). Ordered mesoporous materials for bioadsorption and biocatalysis. Chem Mater.

[23] Shindo T, Kudo H, Kitabayashi S, Ozawa S (2003). Applicability of MCM-41 as column packing in HPLC for the evaluation of aluminum species in partially neutralized aluminum solutions. Microporous and Mesoporous Materials.

[24] Martin T, Galarneau A, Di Renzo F, Brunel D, Fajula F, Heinisch S (2004). Great improvement of chromatographic performance using MCM-41 spheres as stationary phase in HPLC. Chem Mater.

[25] Gustafsson H, Isaksson S, Altskär A, Holmberg K (2016). Mesoporous silica nanoparticles with controllable morphology prepared from oil-in-water emulsions. J Colloid Interface Sci.

[26] Vallet-Regi M, Rámila A, del Real RP, Pérez-Pariente J (2001). A new property of MCM-41:  Drug delivery system. Chem Mater.

[27] Cecilia JA, Moreno Tost R, Retuerto Millán M (2019). Mesoporous materials: From synthesis to applications. Int J Mol Sci.

[28] Kresge CT, Leonowicz ME, Roth WJ, Vartuli JC, Beck JS (1992). Ordered mesoporous molecular sieves synthesized by a liquid-crystal template mechanism. Nature.

[29] Kwon S, Singh RK, Perez RA, Abou Neel EA, Kim HW, Chrzanowski W (2013). Silica-based mesoporous nanoparticles for controlled drug delivery. J Tissue Eng.

[30] Liong M, Lu J, Kovochich M, Xia T, Ruehm SG, Nel AE (2008). Multifunctional inorganic nanoparticles for imaging, targeting, and drug delivery. ACS Nano.

[31] Pang J, Zhao L, Zhang L, Li Z, Luan Y (2013). Folate-conjugated hybrid SBA-15 particles for targeted anticancer drug delivery. J Colloid Interface Sci.

[32] Stöber W, Fink A, Bohn E (1968). Controlled growth of monodisperse silica spheres in the micron size range. J Colloid Interface Sci.

[33] Zhao D, Feng J, Huo Q, Melosh N, Fredrickson GH, Chmelka BF et al (1998). Triblock copolymer syntheses of mesoporous silica with periodic 50 to 300 angstrom pores. Science.

[34] Vallet-Regi M, Balas F, Arcos D (2007). Mesoporous materials for drug delivery. Angew Chem Int Ed Engl.

[35] Slowing II, Vivero-Escoto JL, Wu CW, Lin VS (2008). Mesoporous silica nanoparticles as controlled release drug delivery and gene transfection carriers. Adv Drug Deliv Rev.

[36] Elnaggar YS, El-Massik MA, Abdallah OY (2009). Self-nanoemulsifying drug delivery systems of tamoxifen citrate: Design and optimization. Int J Pharm.

[37] Memisoglu-Bilensoy E, Vural I, Bochot A, Renoir JM, Duchene D, Hincal AA (2005). Tamoxifen citrate loaded amphiphilic beta-cyclodextrin nanoparticles: in vitro characterization and cytotoxicity. J Control Release.

[38] Gao S, Singh J (1998). In vitro percutaneous absorption enhancement of a lipophilic drug tamoxifen by terpenes. J Control Release.

[39] Rao KS, El-Hami K, Kodaki T, Matsushige K, Makino K (2005). A novel method for synthesis of silica nanoparticles. J Colloid Interface Sci.

[40] Wang X, Lin KS, Chan JC, Cheng S (2004). Preparation of ordered large pore SBA-15 silica functionalized with aminopropyl groups through one-pot synthesis. Chem Commun (Camb).

[41] Riva B, Bellini M, Corvi E, Verderio P, Rozek E, Colzani B (2018). Impact of the strategy adopted for drug loading in nonporous silica nanoparticles on the drug release and cytotoxic activity. J Colloid Interface Sci.

[42] Lin JT, Du JK, Yang YQ, Li L, Zhang DW, Liang CL (2017). pH and redox dual stimulate-responsive nanocarriers based on hyaluronic acid coated mesoporous silica for targeted drug delivery. Mater Sci Eng C Mater Biol Appl.

[43] Khosravian P, Shafiee Ardestani M, Khoobi M, Ostad SN, Abedin Dorkoosh F, Akbari Javar H (2016). Mesoporous silica nanoparticles functionalized with folic acid/methionine for active targeted delivery of docetaxel. Onco Targets Ther.

[44] Cheng YJ, Zeng X, Cheng DB, Xu XD, Zhang XZh, Zhuo RX (2016). Functional mesoporous silica nanoparticles (MSNs) for highly controllable drug release and synergistic therapy. Colloids Surf B Biointerfaces.

[45] Shahnani M, Mohebbi M, Mehdi A, Ghassempour A, Aboul-Enein HY (2018). Silica microspheres from rice husk: A good opportunity for chromatography stationary phase. Ind Crops Prod.

[46] Silva CRP, da Rocha Ferreira F, Webler GD, da Silva AOS, de Abreu FC, Fonseca EJS (2017). Encapsulation of mangiferin in ordered mesoporous silica type SBA-15: synthesis and characterization. Mater Res Express.

[47] Barrett EP, Joyner LG, Halenda PP (1951). The determination of pore volume and area distributions in porous substances I Computations from nitrogen isotherms. J Am Chem Soc.

[48] He Y, Liang S, Long M, Xu H (2017). Mesoporous silica nanoparticles as potential carriers for enhanced drug solubility of paclitaxel. Mater Sci Eng C Mater Biol Appl.

[49] Mortazavi Y, Ghoreishi SM (2016). Synthesis of mesoporous silica and modified as a drug delivery system of ibuprofen. J Nanostruct.

[50] Li T, Shen X, Geng Y, Chen Z, Li L, Li S (2016). Folate-functionalized magnetic-mesoporous silica nanoparticles for drug/gene codelivery to potentiate the antitumor efficacy. ACS Appl Mater Interfaces.

[51] Goel S, Mishra P (2019). Thymoquinone loaded mesoporous silica nanoparticles retard cell invasion and enhance in vitro cytotoxicity due to ROS mediated apoptosis in HeLa and MCF-7 cell lines. Mater Sci Eng C Mater Biol Appl.

[52] Ahmadi E, Dehghannejad N, Hashemikia S, Ghasemnejad M, Tabebordba H (2014). Synthesis and surface modification of mesoporous silica nanoparticles and its application as carriers for sustained drug delivery. Drug Deliv.

[53] Chen SY, Huang CY, Yokoi T, Tang CY, Hung SG, Lee JJ (2012). Synthesis and catalytic activity of amino-functionalized SBA-15 materials with controllable channel lengths and amino loadings. J Mater Chem.

[54] Lou F, Zhang G, Ren L, Guo X, Song Ch (2021). Impacts of nano-scale pore structure and organic amine assembly in porous silica on the kinetics of CO2 adsorptive separation. Nano Res.

[55] Liou TH, Chen GW, Yang S (2022). Preparation of amino-functionalized mesoporous SBA-15 nanoparticles and the improved adsorption of tannic acid in wastewater. Nanomaterials (Basel).

[56] Zhang Y, Qian C, Duan J, Liang Y, Luo J, Han Y (2022). Synthesis of HKUST-1 embedded in SBA-15 functionalized with carboxyl groups as a catalyst for 4-nitrophenol to 4-aminophenol. Appl Surf Sci.

[57] Ehsanimehr S, Najafi Moghadam P, Dehaen W, Shafiei-Irannejad V (2021). PEI grafted Fe3O4@SiO2@SBA-15 labeled FA as a pH-sensitive mesoporous magnetic and biocompatible nanocarrier for targeted delivery of doxorubicin to MCF-7 cell line Colloids and Surfaces A. Physicochemical and Engineering Aspects.

[58] Lestari WA, Wahyuningsih S, Gomez-Ruiz S, Wibowo FR (2022). Drug loading ability and release study of various size small mesoporous silica nanoparticle as drug carrier. J Phys Conf Ser.

